# miRNA expression profiles of peripheral white blood cells from beef heifers with varying reproductive potential

**DOI:** 10.3389/fgene.2023.1174145

**Published:** 2023-05-10

**Authors:** Priyanka Banerjee, Wellison J. S. Diniz, Soren P. Rodning, Paul W. Dyce

**Affiliations:** Department of Animal Sciences, Auburn University, Auburn, AL, United States

**Keywords:** beef heifer, miRNA, pathways, reproductive potential, small-RNA sequencing

## Abstract

Reproductive performance is the most critical factor affecting production efficiency in the cow-calf industry. Heifers with low reproductive efficiency may fail to become pregnant during the breeding season or maintain a pregnancy. The cause of reproductive failure often remains unknown, and the non-pregnant heifers are not identified until several weeks after the breeding season. Therefore, improving heifer fertility utilizing genomic information has become increasingly important. One approach is using microRNAs (miRNA) in the maternal blood that play an important role in regulating the target genes underlying pregnancy success and thereby in selecting reproductively efficient heifers. Therefore, the current study hypothesized that miRNA expression profiles from peripheral white blood cells (PWBC) at weaning could predict the future reproductive outcome of beef heifers. To this end, we measured the miRNA profiles using small RNA-sequencing in Angus-Simmental crossbred heifers sampled at weaning and retrospectively classified as fertile (FH, *n* = 7) or subfertile (SFH, *n* = 7). In addition to differentially expressed miRNAs (DEMIs), their target genes were predicted from TargetScan. The PWBC gene expression from the same heifers were retrieved and co-expression networks were constructed between DEMIs and their target genes. We identified 16 differentially expressed miRNAs between the groups (*p*-value ≤0.05 and absolute (log2 fold change ≥0.05)). Interestingly, based on a strong negative correlation identified from miRNA-gene network analysis with PCIT (partial correlation and information theory), we identified miRNA-target genes in the SFH group. Additionally, TargetScan predictions and differential expression analysis identified bta-miR-1839 with *ESR1* **,** bta-miR-92b with *KLF4* and *KAT2B*, bta-miR-2419-5p with *LILRA4*, bta-miR-1260b with *UBE2E1*, *SKAP2* and *CLEC4D*, and bta-let-7a-5p with *GATM*, *MXD1* as miRNA-gene targets. The miRNA-target gene pairs in the FH group are over-represented for MAPK, ErbB, HIF-1, FoxO, p53, mTOR, T-cell receptor, insulin and GnRH signaling pathways, while those in the SFH group include cell cycle, p53 signaling pathway and apoptosis. Some miRNAs, miRNA-target genes and regulated pathways identified in this study have a potential role in fertility; other targets are identified as novel and need to be validated in a bigger cohort that could help to predict the future reproductive outcomes of beef heifers.

## 1 Introduction

Reproductive traits are essential for sustainable food production. Low reproductive capacity in beef heifers is inferred as a failure to become pregnant during the breeding season or maintain a pregnancy to calving (Lamb, 2013). The possible causes for reproductive failure in heifers may be attributed to poor reproductive and nutritional management, diseases, and genetics (Houghton et al., 1990; BonDurant, 2007; Larson and White, 2016; Shao et al., 2021). To overcome this problem, producers often develop more heifers than required for replacements and perform additional inseminations or hormonal treatments that consequently alter the current and subsequent cow-calf production ratio (Shao et al., 2021). In addition to the cost of artificial insemination (AI) and treatments, losses are further increased due to the rearing of the animal from birth to breeding age and then culling and replacing the animals with poor reproductive performance. Management practices to select heifers with high reproductive potential contribute to increasing efficiency; however, some heifers still fail to conceive. Considering this, the beef production sector is particularly interested in identifying biomarkers to predict reproductive efficiency.

In recent years, developments in molecular biology have provided new insights into the potential candidates and biomarkers for fertility and predicting reproductive outcomes. For instance, Binelli et al. (2015) identified transcriptome signatures in uterine biopsies from pregnant cows at 6 days post-AI in beef cattle. Forde et al. (2012) identified increased gene expression in the endometrium of pregnant cattle. Machine learning approaches have provided opportunities to unravel genomic signatures for fertility from omics data. These approaches are powerful in processing and modeling omics data with vast and diverse volumes (Li et al., 2022). Among the studies, Rabaglino and Kadarmideen, (2020) identified 50 genes from endometrial transcriptome as predictors of uterine receptivity to embryo transfer in cattle. Likewise, Diniz et al. (2022) reported nine potential candidates using a multi-tiered approach of machine learning and gene co-expression network on the transcriptome profile of uterine luminal epithelial cells. Although these studies provided insights into the different expression levels of potential candidates regulating fertility or reproductive outcome, the downstream molecular targets regulating the expression of these genes were not investigated. They were also unable to predict reproductive potential at weaning as they were conducted at or near the time of insemination.

Among the factors regulating gene expression, a class of RNA molecules, microRNAs (miRNAs), are known to regulate gene expression. Such regulation is mainly based on binding with messenger RNA (mRNA) targets and destabilizing them, thereby repressing protein production and translational silencing (Cannell et al., 2008). A plethora of studies identified miRNAs as biomarkers for pregnancy outcomes in humans (Barchitta et al., 2017; Ali et al., 2021; Vashukova et al., 2021; Xu et al., 2021) and cattle (Ioannidis and Donadeu, 2016; De Bem et al., 2017; Pohler et al., 2017; Gebremedhn et al., 2018; Lim H.-J. et al., 2021). Ioannidis and Donadeu reported an increased level of circulating miR-26a on days 16–24 of pregnancy in cattle (Ioannidis and Donadeu, 2016). Similarly, levels of circulating miR-221 and miR320a were increased in weeks 8, 12 and 16 of pregnancy in cattle (Lim H.-J. et al., 2021).

Collectively, these findings provide critical insights into the biological mechanisms determining different reproductive outcomes. However, most of the studies have focused on time periods in proximity to the breeding season. Considering the producer has invested time and resources in developing the replacement heifers, using these potential biomarkers at breeding is too late. Thus, it would be beneficial to trace back the potential biomarkers through weaning—when the selection of replacement animals usually takes place. Therefore, our main objective was to profile the miRNA levels in the peripheral white blood cells (PWBC) of beef heifers at weaning that could potentially be used to predict heifers with a high reproductive outcome following AI. We hypothesized that the miRNAs are differentially expressed, the genes regulated by these miRNAs are co-expressed, and the miRNA-gene networks are rewired in beef heifers at an early development stage, contributing to a varying reproductive outcome at maturity.

## 2 Materials and methods

### 2.1 Animal handling, sample collection, phenotyping, and classification

All procedures involving animals were approved by the Institutional Animal Care and Use Committee (IACUC) at Auburn University (IACUC protocol numbers 2015-2786 and 2019-3591). Cross-bred heifers (Angus-Simmental, *n =* 75) utilized in this study originated from and were housed at an outlying Alabama agricultural experiment station research and extension center (Auburn University). The heifers were weaned ∼238 days after birth. Phenotypic data, such as weaning age and weight, were recorded for each heifer. Immediately after weaning, 10 mL of blood was drawn into ethylenediaminetetraacetic acid (EDTA) vacutainers (Becton, Dickinson and Company, Franklin, NJ) from the jugular vein. The vacutainers were inverted 8–10 times and immersed in ice until immediate processing. In the laboratory, blood was processed to isolate peripheral white blood cells (PWBC), as described elsewhere (Banerjee et al., 2023). The PWBCs were stored at −80°C until further processing.

**FIGURE 1:**
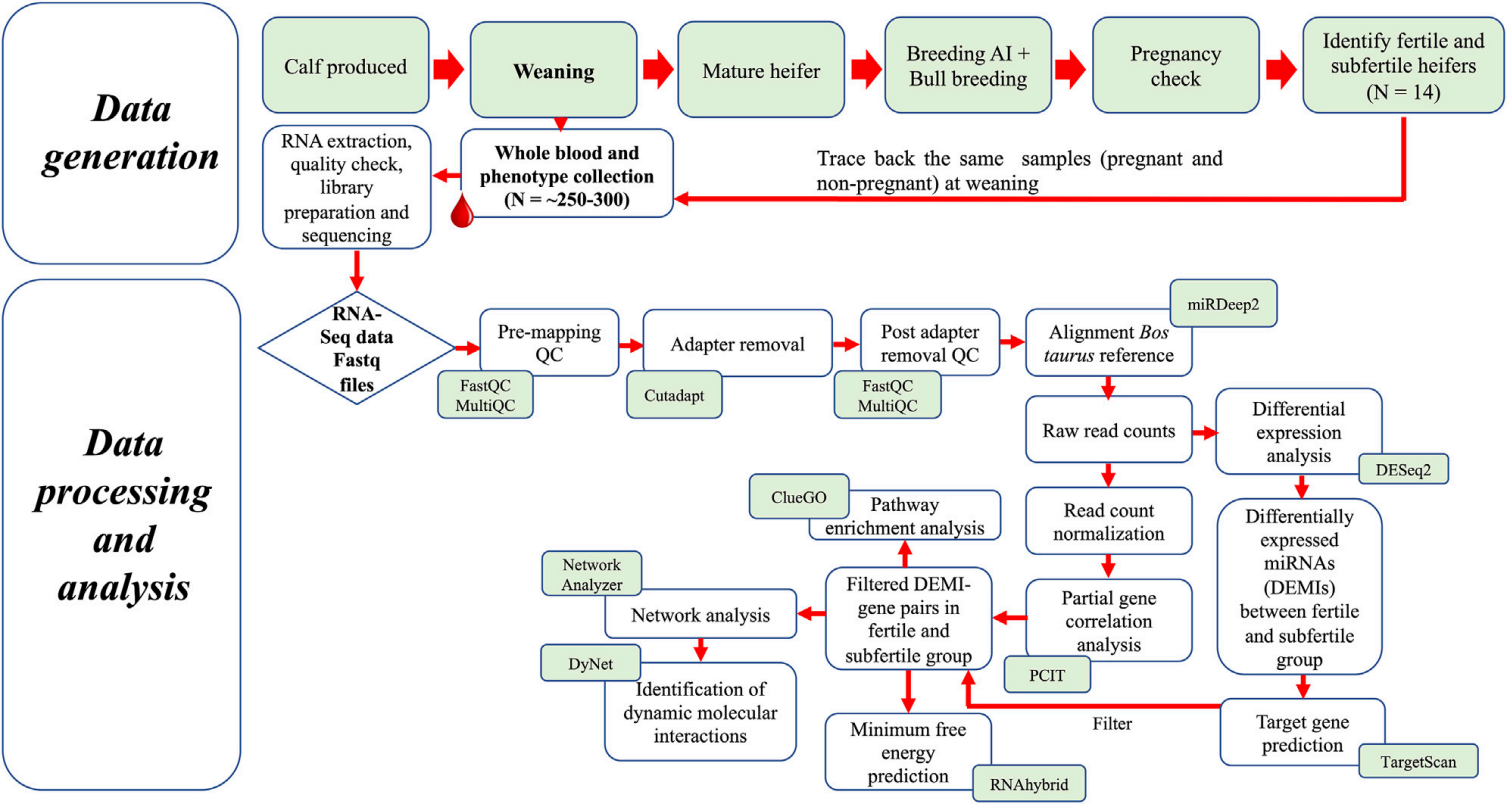
Schematic representation of the study design and analysis steps.

For breeding, 72 heifers were selected based on ideal body condition scores (5–6) and reproductive tract scores (≥4). The breeding protocol, estrus synchronization and fixed-time artificial insemination (AI) have been described previously (Banerjee et al., 2023). In brief, approximately 45 days before breeding by AI, pre-breeding examinations for each heifer were performed to evaluate the pubertal status. All heifers underwent an estrus synchronization and fixed-time AI protocol (7-Day CO-Synch + CIDR) (Dickinson et al., 2018). Fourteen days following AI, all heifers were exposed to fertile bulls for a 60-day natural service breeding season to ensure adequate opportunities for conception to occur. Pregnancy of the heifers was evaluated on day 75 post-AI by transrectal palpation and ultrasound. The presence and gestation age or absence of conceptus was confirmed 75 days following AI with ultrasound and transrectal palpation and was used to classify heifers as fertile to those that were pregnant to AI (FH, *n =* 35), pregnant to natural breeding (P-NB, *n =* 26) or subfertile that were not pregnant (SFH, *n =* 11). Heifers that were pregnant to AI (FH, *n =* 7) and subfertile (SFH, *n =* 7) and with comparable birth age, weaning age and body weight in both groups were selected for this study. A schematic representation of the study design and analysis steps is given in Figure 1.

### 2.2 RNA extraction from PWBC

Based on the pregnancy classification of heifers, total RNA was extracted from the 14 PWBC samples (FH and SFH) that were collected at the time of weaning. The RNA was extracted using the Trizol reagent (Invitrogen, Carlsbad, CA, United States) following the manufacturer’s protocol. Additional RNA purification and DNase digestion steps using an RNA clean and concentrator kit (Zymo Research, Irvine, CA, United States) were included in the protocol. RNA was quantified on a Qubit fluorometer using a Qubit RNA broad-range assay kit (Life Technologies, Thermo Fisher Scientific Inc., MA, United States). The RNA integrity was assessed using the Agilent Bioanalyzer and the Agilent RNA 6000 Nano kit (Agilent, Santa Clara, CA, United States). The quality of small RNAs was determined using the Agilent 2100 Bioanalyzer Small RNA kit (Agilent, Santa Clara, CA, United States). The samples with average RNA integrity number (RIN) values >6.8 were further processed for small RNA library construction.

### 2.3 Library preparation and sequencing

The total RNA of each sample was diluted with RNase-free water to obtain a final concentration of 1 μg as a starting material. This diluted total RNA was used to prepare libraries using the protocols by NEXTflex small RNA-Seq kit v3 (Perkin Elmer). Following the protocol from the kit, the 5′ and 3’ adapters were ligated to the RNA fragments, which were then reverse-transcribed and amplified (18 cycles) to generate cDNA libraries. Each cDNA library was prepared using a different barcode primer for the ease of being multiplexed for sequencing. Libraries were cleaned using NEXTflex Cleanup beads (gel-free protocol), and the size distribution of the final library was assessed by Agilent Bioanalyzer high-sensitivity DNA assay (Agilent, Santa Clara, CA, United States). The quality-checked libraries were pooled and sequenced in the NextSeq 500 using the single-end 50 bp chemistry at Discovery Life Sciences (Hudson Alpha Institute of Biotechnology, Huntsville, AL, United States).

### 2.4 Data processing and miRNA expression profiling

Raw sequence demultiplexed reads obtained from Hudson Alpha were initially assessed for sequencing quality using FastQC v0.11.9 (Andrew, 2010) and MultiQC v1.12 (Ewels et al., 2016). The reads were evaluated for quality based on average read length, adaptor content, per-sequence GC content, and sequence quality scores. After that, the 3′ adapter sequence was trimmed using Cutadapt (Martin, 2011) with the following parameters: *-a* TGG​AAT​TCT​CGG​GTG​CCA​AGG -*minimum length 23*. The reads were further trimmed using Cutadapt (Martin, 2011) to remove four bases from either side of each read following the small RNA trimming instructions (cutadapt -u 4 -u -4) (recommended by NEXTflex small RNA-Seq kit v3 (Perkin Elmer)). Trimmed Fastq files were checked for quality control with FastQC v0.11.9 and MultiQC v1.12. To profile both novel and known miRNA expression in the samples from the cleaned sequence data, the trimmed reads were processed using the miRDeep2 analysis workflow (Friedländer et al., 2012). Sequences were aligned to Ensembl’s ARS-UCD 1.2 *Bos taurus* reference genome (https://useast.ensembl.org/Bos_taurus/Info/Index) using the mapper.pl module in miRDeep2 and were further aligned with *B. taurus* precursor and matured miRNAs extracted from miRBase v22.1 (Kozomara et al., 2019). Only the alignments with zero mismatches in the seed region and those greater than 18 nt of a read mapped to the genome were retained, producing read counts for each sample.

### 2.5 Differential expression analysis

Initially, the mature miRNAs with zero counts were filtered out. Thereafter, the read counts were transformed to counts per million (CPM) using edgeR v3.28.1 (Robinson et al., 2010). For quality control, raw counts with CPM <1 in 50% of the samples were filtered out. To identify the differentially expressed miRNAs, the raw counts were analyzed in DESeq2 v1.26.0 (Love et al., 2014). The differentially expressed miRNAs (DEMIs) identified with a *p*-value ≤0.05 and absolute (log2 fold change) ≥ 0.5 were considered significant. The DEMIs were classified as up or downregulated based on the sign of log2 fold change in the SFH group. The up and downregulated miRNAs were visualized using a volcano plot constructed using the R-package EnhancedVolcano v1.4.0 (Blighe et al., 2018).

### 2.6 Target gene prediction

The target genes for DEMIs were predicted using TargetScan v8.0, which predicts the targets of miRNAs by searching the conserved 8mer, 7mer and 6mer sites that match the seed region of each miRNA (Lewis et al., 2005). The search for mRNA targets was specific to *B. taurus.* As a feature of TargetScan, the predictions were ranked based on targeting efficacy estimated using cumulative weighted context++ scores (Agarwal et al., 2015). The genes with high confidence (≤−0.4) in the cumulative weighted context++ score were selected as target genes for each miRNA (Agarwal et al., 2015).

### 2.7 mRNA-miRNA co-expression and network analysis

To identify the correlation pattern between the DEMI-gene targets identified by TargetScan, we also generated PWBC mRNA expression profiles from the same heifers as reported elsewhere (Banerjee et al., 2023). In brief, the mRNA profile of the same set of samples from FH (*n* = 7) and SFH (*n* = 7) was sequenced to generate paired-end 100 bp reads. After a quality check using FastQC v0.11.9 and MultiQC v1.12, the raw sequences were mapped using STAR aligner v2.7.5 (Dobin et al., 2013) to Ensemble’s ARS-UCD1.2 *B. taurus* genome as the reference. The raw counts per gene (obtained using the -*quantMode* function in STAR) were transformed to CPM using edgeR v3.28.1. The genes identified from the PWBC mRNA expression profiles were filtered to retain only those identified as miRNA targets based on TargetScan and were named ‘PWBC expressed TargetScan genes’. To determine the miRNA-gene correlation, we used the partial correlation and information theory (PCIT) approach. The CPM normalized ‘PWBC expressed TargetScan genes’ and DEMIs were used as an input for PCIT. The PCIT reports the significantly correlated pairs after comparing the possible trios of genes (Reverter and Chan, 2008), which allows us to build co-expression networks.

The networks were constructed using the correlations identified by PCIT between DEMIs and ‘PWBC-expressed TargetScan genes’ for each FH and SFH group in Cytoscape v3.8.2 (Shannon et al., 2003). The network for each group was analyzed using the Network Analyzer tool in Cytoscape v3.8.2 (Assenov et al., 2008). Next, to determine the differentially connected genes and miRNAs in each group, the network connectivity (K) measure for each network was standardized by taking a ratio of gene connectivity (degree) and maximum connectivity in each network. The differential connectivity (DK) measure was calculated as $DK_{i}=KSFH_{\left(i\right)}-KFH_{\left(i\right)}$ . The DK values were transformed to a z-score and ±1.96 SD was considered significant (*p* ≤ 0.05). The connectivity gain or loss was evaluated for each gene or miRNA in the SFH group as the reference. The central reference network comparing both groups was constructed using DyNet (Cytoscape plug-in) (Goenawan et al., 2016). DyNet allows for identifying and visualizing nodes and edge rewiring in response to cellular signals and highlights the most rewired nodes based on the central reference network constructed with FH and SFH groups (Goenawan et al., 2016).

### 2.8 Minimum free energy for miRNA-target prediction

To cross-validate the specificity of the target genes identified from TargetScan and correlate to the DEMIs, we used the RNAhybrid (Rehmsmeier et al., 2004). To this end, we retrieved the mature miRNA sequence from the miRBase database (Kozomara et al., 2019) and the cDNA sequence of the genes from BioMart (Durinck et al., 2005). The minimum free energy (mfe) was predicted for the miRNA-mRNA sequence; several miRNAs predicted to have a low mfe indicate a relatively high affinity for miRNA-mRNA complexes. The cutoff for mfe was set to less than −20 kcal/mol for evaluating miRNA-mRNA affinity (Yen et al., 2019; de Lima et al., 2021). Average mfe was calculated for the genes with multiple transcripts.

### 2.9 Functional enrichment analysis

The pathway over-representation analysis for the genes correlated with miRNAs identified by PCIT was carried out using ClueGO v2.5.8 (Cytoscape plug-in) (Bindea et al., 2009). The redundant terms were clustered based on a kappa score of 0.4, and the *B. taurus* annotation was used as the background in ClueGO. The significant pathways were selected based on the corrected *p*-value with Bonferroni step-down feature ≤0.05.

## 3 Results

### 3.1 Data generation and mapping statistics

We used small RNA-Seq to identify the difference in the expression of miRNAs from PWBC between two groups, FH and SFH. The sequencing from all the samples yielded an average of 6.9 million reads. On average, 78.13% of reads were uniquely mapped to the *B. taurus* reference genome (Supplementary Table S1), yielding a dataset of 1,207 miRNAs in 14 samples. Following filtering to remove the zero counts, 614 miRNAs were retained. After the CPM filter approach, 341 miRNAs in 14 samples were used for further analysis.

### 3.2 Differentially expressed miRNAs (DEMIs)

**FIGURE 2:**
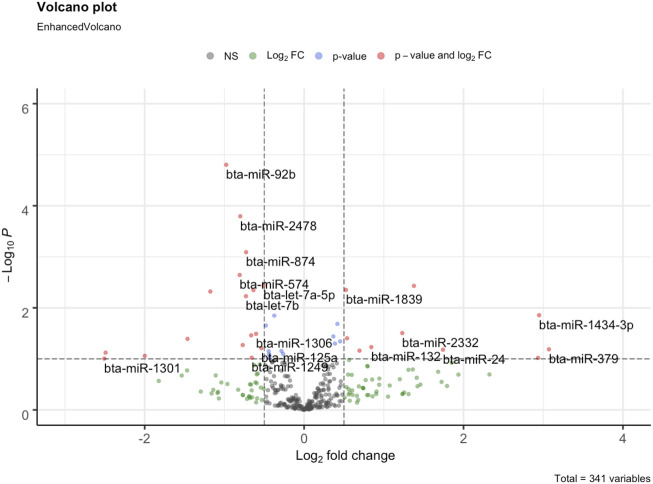
Volcano plot of differentially expressed miRNAs between FH and SFH groups. Each dot corresponds to a miRNA. The log2fold change is represented in the *x*-axis represents, while the negative log (base 10) of the *p*-value in the *y*-axis. The horizontal dashed line represents the threshold with a *p*-value cutoff <0.05, while the vertical bars represent the absolute log2fold change >0.5. The 16 DEMIs are labeled in the plot. The left panel (0 to −3 of log2fold change) is downregulated, while the right panel (0–4 of log2fold change) is upregulated miRNAs.

We identified 16 DEMIs between the FH and SFH group out of 341 miRNAs [(*p*-value ≤0.05 and absolute (log2 fold change ≥0.5)] (Figure 2; Supplementary Table S2). The top 5 upregulated miRNAs in the SFH group with the highest fold change difference included bta-miR-677, bta-miR-1839, bta-miR-1434-3p, bta-miR-2332 and bta-miR-140, while the downregulated miRNAs were bta-miR-450b, bta-miR-2419-5p, bta-miR-92b, bta-miR-574 and bta-miR-2478.

### 3.3 Target gene prediction and overlapping with genes expressed in PWBC

**FIGURE 3:**
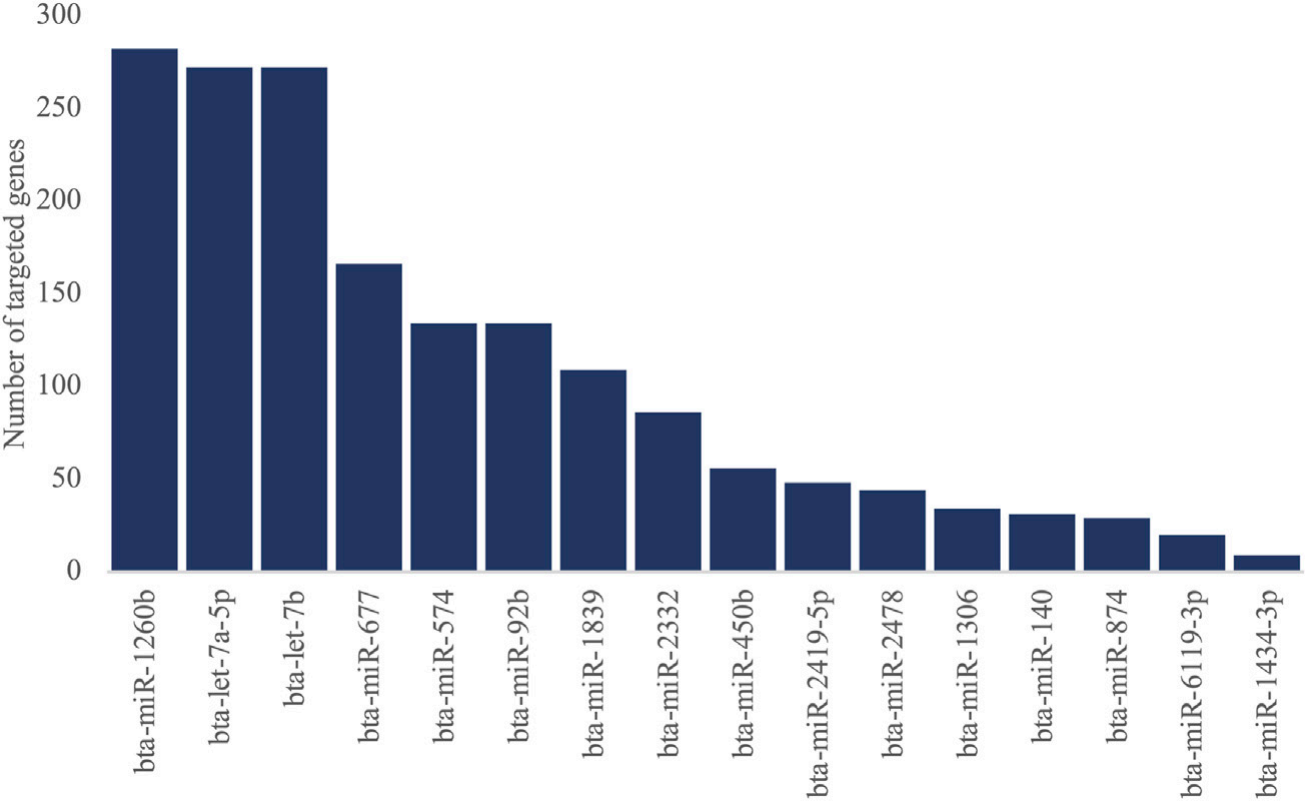
Number of genes targeted by 16 DEMIs (ranked in descending order).

The predicted gene targets for 16 DEMIs were retrieved from TargetScan. The genes with high confidence (≤−0.4) in the cumulative weighted context++ score were selected as target genes for each miRNA. We identified 1,365 genes targeted by the 16 DEMIs (Figure 3), out of which 741 were expressed in PWBC (Supplementary Table S3). Out of the 741 genes, nine were previously reported as differentially expressed in PWBC from the same set of FH and SFH heifers (Supplementary Table S3) (Banerjee et al., 2023). The nine genes and their miRNA targets are as follows: bta-miR-92b targeted *KLF4* and *KAT2B*, bta-miR-2419-5p targeted *LILRA4*, bta-miR-1260b targeted *UBE2E1*, *SKAP2* and *CLEC4D*, bta-let-7a-5p targeted *GATM*, *MXD1* and bta-miR-1839 targeted *ESR1*.

### 3.4 miRNA-mRNA co-expression and network analysis

**FIGURE 4:**
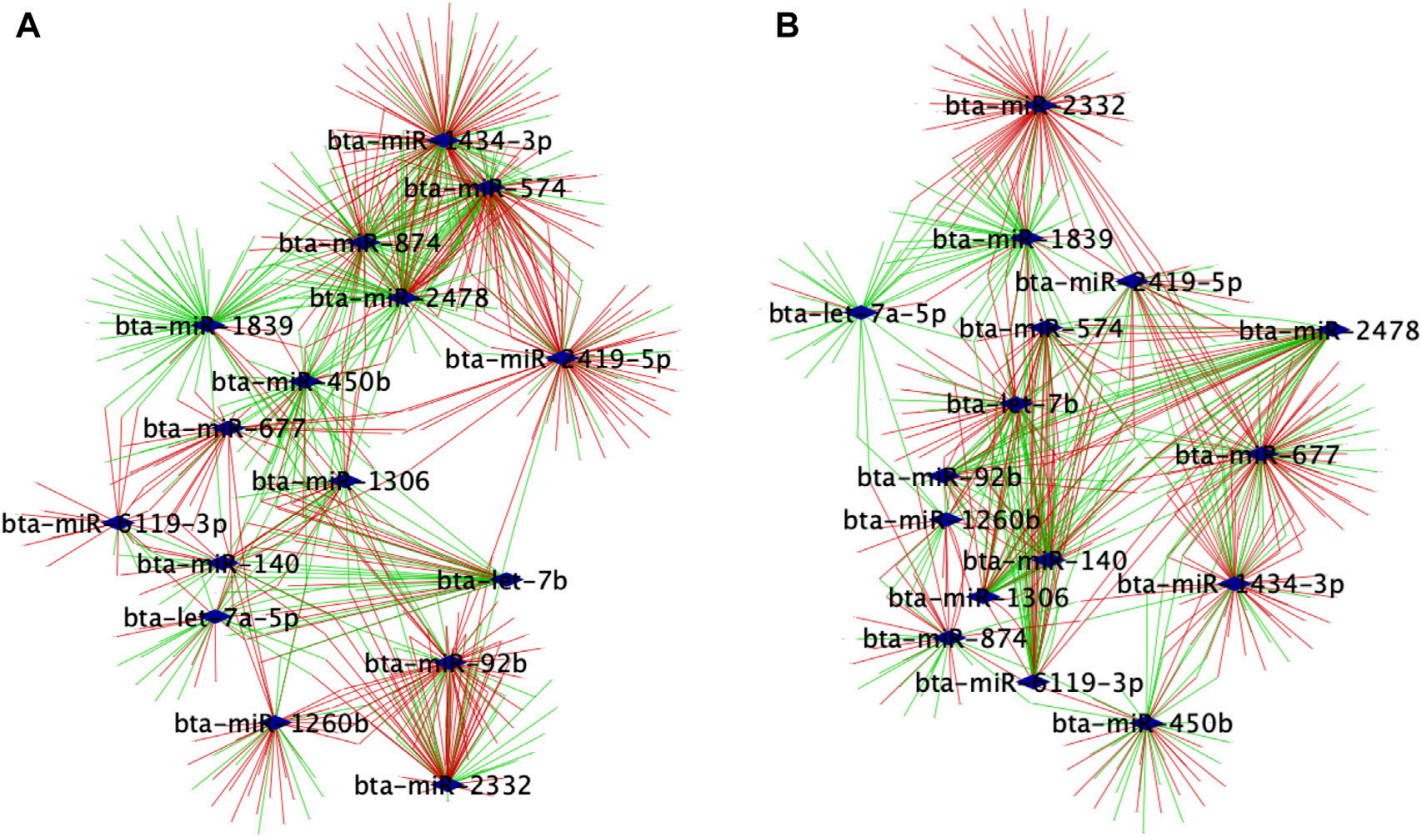
Regulatory networks of co-expressed genes with 16 DEMIs in **(A)** FH and **(B)** SFH groups. Nodes are genes significantly correlated with miRNAs, while edges are positive or negative interactions (correlations) between the miRNA and target genes. The blue diamond represents the DEMIs; green strokes represent positive correlations, while red strokes represent negative correlations.

**FIGURE 5:**
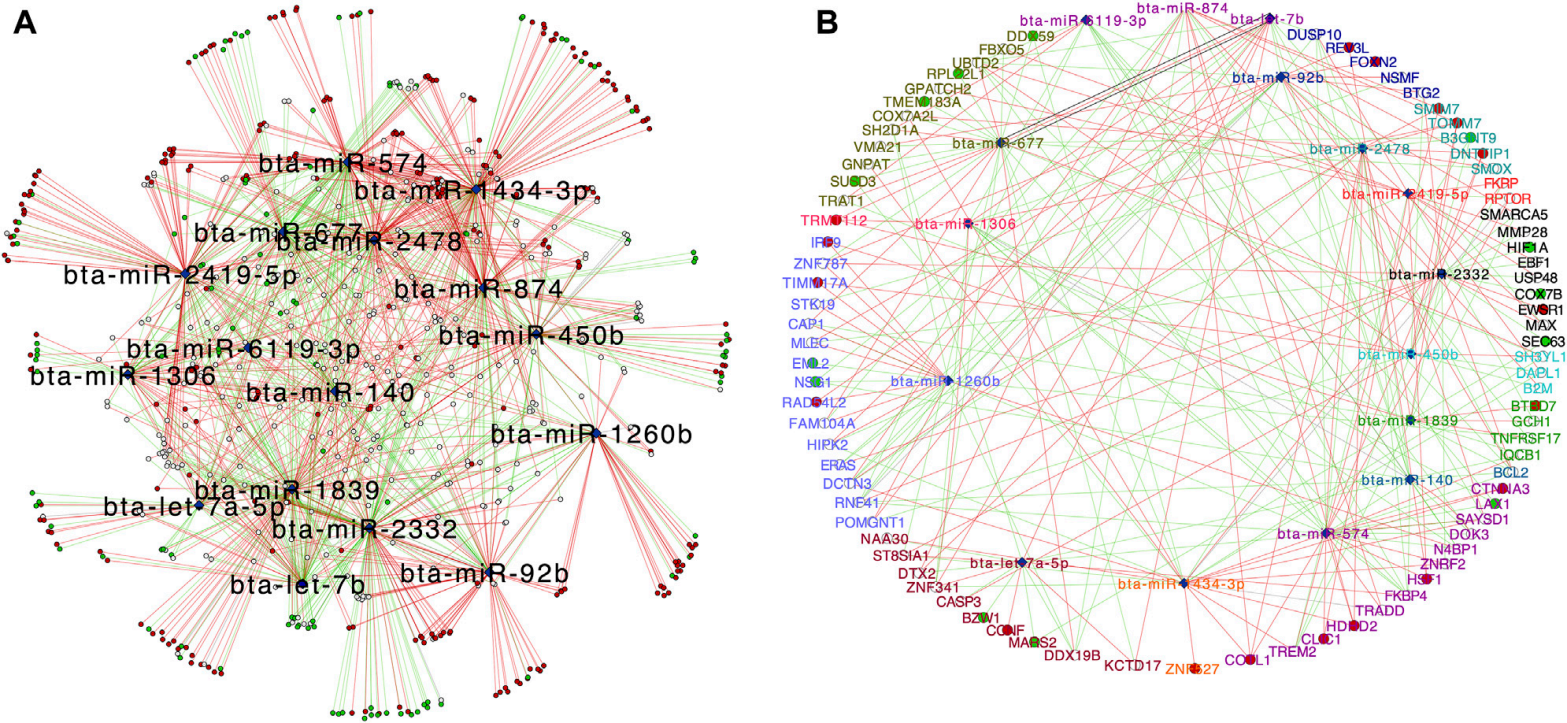
Central reference network constructed using DyNet. **(A)** Network comparison based on the rewired node in the FH and SFH group. The network comprises of 650 nodes and 1547 edges. The blue diamond represents the DEMIs. Unique nodes are shown in red (FH) and green (SFH). Shared nodes are shown in white. **(B)** Central reference network showing the miRNA-gene correlated pair as identified from TargetScan (Supplementary Table S7). Unique nodes are shown in red (FH) and green (SFH). Shared nodes are shown in white. For ease of visualization, each miRNA-target gene pair is labeled with the same color.

We used the PCIT algorithm to build two networks (each from FH and SFH groups) to identify the coordinated expression patterns and potential regulatory role between genes and miRNAs. For the network, the correlation of CPM normalized PWBC-expressed TargetScan genes (*n* = 741) (see Methods) and DEMI (*n* = 16), corresponding to 7 samples in FH and 6 in the SFH group, was used. One sample was removed from the SFH group due to the poor quality of the mRNA sequence. We identified 33,014 and 29,280 correlations in FH and SFH groups, respectively, consisting of miRNA-gene, miRNA-miRNA, and gene-gene correlations (Supplementary Table S4). To retrieve the biologically meaningful miRNAs and reduce data dimensionality, we only retrieved the miRNA-miRNA and miRNA-gene pairs (Supplementary Table S5). We identified 926 and 689 significantly correlated pairs (*r* > |0.6|, *p* ≤ 0.05) in the FH and SFH groups (corresponding to 546 and 409 unique genes) (Supplementary Table S5). The network for each group was visualized using Cytoscape (Figures 4A, B). Of the 16 DEMIs, 15 miRNAs were differentially connected with only bta-miR-1260b not having differential connections. Overall, the SFH group exhibited a loss of miRNA-mRNA network connectivity (Supplementary Table S6). Next, we identified the correlation pattern of the miRNA-gene pair in our study that was identified from TargetScan (Supplementary Table S7). Of the 741 predicted target genes with the corresponding miRNA, 48 and 39 genes were correlated with 12 and 11 DEMIs in the FH and SFH groups, respectively (Supplementary Table S7; Figures 5A, B). Out of these pairs, we identified 30 and 27 negative correlations among the miRNA-gene pairs in FH and SFH, respectively (Supplementary Table S7). Some correlated pairs included bta-miR-2478 with *SMIM7*, bta-miR-574 with *TRADD*, *HDHD2* and *HSF1*, and bta-miR-2332 with *MAX* in the FH group; and bta-miR-1260b with *ERAS*, bta-miR-2332 with *SEC63* and *HIF1A* in the SFH group, exhibited a high negative correlation (*r* = 0.9). All the miRNA-gene pairs exhibited a mfe <−20 kcal/mol, except bta-miR-450b with *SH3YL1* (−18.9 kcal/mol) and with *B2M* (−19.7 kcal/mol).

### 3.5 Pathway analysis

To translate the genes targeted by the miRNAs from PCIT into biologically meaningful processes, we performed pathway enrichment analysis. The top significant (Bonferroni corrected Group *p*-value ≤0.05) KEGG pathways identified in the FH group include MAPK, ErbB, HIF-1, FoxO, p53, mTOR, T-cell receptor, insulin and GnRH signaling, apoptosis and pathways regulating pluripotency of stem cells (Supplementary Table S8). The top significant KEGG pathways of the SFH group include cell cycle, p53 signaling pathway and apoptosis (Supplementary Table S9). Additionally, the biological process in the SFH group includes a hormone-mediated signaling pathway, cellular response to stress, negative regulation of growth, inflammatory response to antigenic stimulus and signal transduction in response to DNA damage (Supplementary Table S9).

## 4 Discussion

Evidence suggests that miRNAs play an essential regulatory role in several biological processes involving cell proliferation, cell death, epigenetic changes, and apoptosis (Bartel, 2004; Cupp et al., 2009). All these processes have the potential to promote phenotypic variation within the population. Among the traits, phenotypes associated with reproductive outcome and fertility are of major interest to cattle producers, mainly because of the high production loss from a heifer of low reproductive potential. Specifically in reproductive biology, miRNAs have been demonstrated to be important regulators of embryonic development in humans and livestock, such as predicting pregnancy outcomes (Ioannidis and Donadeu, 2016; Hitit et al., 2022), embryo viability (Pohler et al., 2017) and implantation (Kose et al., 2022), and endometrial receptivity (Zang et al., 2021). Moreover, miRNAs regulate the gene targets by repressing or stimulating their expression (Orang et al., 2014). Thus, the present study was undertaken to identify the difference in the miRNA expression and the respective gene targets in the PWBC of beef heifers at weaning and their potential as predictors of future reproductive potential in beef heifers.

We identified five miRNAs (bta-miR-92b, bta-miR-2419-5p, bta-miR-1260b, bta-miR-1839 and bta-let-7a-5p) targeting differentially expressed genes from PWBC of the same beef heifers. Among them, bta-miR-92b is the most significant miRNA downregulated in the SFH group. Expression of miR-92b was significantly upregulated in the porcine placenta on day 90 of gestation (Su et al., 2010). In a study reported in cattle, miR-92b (downregulated expression) was associated with endometritis, an inflammatory response in the endometrium that causes reproductive disorder (Jiang et al., 2021). The authors further reported that over-expression of miR-92b significantly suppressed the activation of the toll-like receptor in lipopolysaccharide-mediated bovine endometrial cells, thereby reducing pro-inflammatory cytokines and inhibiting cell apoptosis (Jiang et al., 2021). Interestingly, in a separate study, miR-92 has been identified to regulate *KLF4* (Hamik and Jain, 2012). In female fertility, *KLF4* mediates the anti-proliferative effects of progesterone during the G0/G1 arrest in endometrial epithelial cells in humans (Shimizu et al., 2010). In our study, *KLF4* was identified as one of the potential targets of bta-miR-92b from TargetScan and associated with pathways involved with post-embryonic development, cell-cell adhesion, and immune system development in the FH group. Another target gene associated with bta-miR-92b in our study was *KAT2B*. With the pathway analysis, we identified *KAT2B* underlying negative regulation of serine/threonine kinase activity and phosphorylation pathways in the FH group. Interestingly, *KLF4* and *KAT2B* have been identified as downregulated in the PWBC of non-pregnant heifers compared to pregnant heifers (Banerjee et al., 2023). Furthermore, from the PCIT network analysis, we identified bta-miR-92b as more connected with genes/miRNA targets in fertile heifers (68 targets) than in subfertile heifers (31 targets). Among the common genes from TargetScan and PCIT, we found that the bta-miR-92b was correlated with the *BTG2*, *REV3L*, *FOXN2* and *NSMF* genes in the FH group and *DUSP10* in the SFH group. *DUSP10* is associated with negative regulation of growth, phosphorylation, and transferase and kinase activity. *DUSP10* plays a significant role in the innate and adaptive immune response by regulating the mitogen-activated protein (MAP) kinase modulated by kinase and phosphatase (Zhang et al., 2004). Despite these findings, further mechanisms of bta-miR-92b and its gene targets need to be explored for fertility and reproductive outcome in bovines.

Bta-miR-1260b was significantly downregulated in the SFH group. In previous studies, miR-1260 has been associated with infertility in humans (Butler et al., 2021). From TargetScan, we identified *UBE2E1*, *SKAP2* and *CLEC4D* as predicted genes targeted by bta-miR-1260b. These genes were differentially expressed in the PWBC of beef heifers at weaning (Banerjee et al., 2023). *SKAP2* and *CLEC4D* were associated with regulating the immune response pathway in the FH group. *SKAP2* is a substrate of Src family kinase and regulates cellular processes, including proliferation, adhesion, migration and stress response (He et al., 2017). In a previous study, *SKAP2* was detected in all developmental stages of mouse oocytes and depletion of *SKAP2* caused the failure of spindle migration, polar body extrusion and cytokinesis defects (He et al., 2017). In a separate study in sheep, *SKAP2* was differentially expressed in the granulosa cells from superstimulated lamb and ewe follicles and was associated with cellular growth, proliferation, and migration (Wu et al., 2016). *CLEC4D* was found to play important roles in immunity and homeostasis (Graham et al., 2012). Besides *SKAP2* and *CLEC4D*, bta-miR-1260b had a high correlation (*r* > |0.9|) with *ERAS* and *NSG1* in the SFH group, identified from the network analysis. Furthermore, *ERAS* was identified with a mfe of −30.5 kcal/mol confirming as a predictive target of bta-miR-1260b. In bovines, lipopolysaccharide treatment of bovine endometrial epithelial cells caused differential methylation of the *NSG1* gene associated with inflammation and endometrial function (Jhamat et al., 2020). These studies suggest the role of the genes targeted by bta-miR-1260b with inflammation and immune response. Inflammation and immune response are connected at many levels with fertility and reproductive outcome in cattle (Fair, 2015); however, the detailed mechanisms still need to be explored.

Bta-miR-1839 was upregulated but had less miRNA-gene network connections (64 targets) in the SFH group compared to the FH group (69 targets). In a microarray-based mice study, an increase in the expression of miR-1839 was observed during the implantation periods (day 5) in the luminal epithelium and endometrium (Li et al., 2015). We identified *ESR1* (estrogen receptor 1) targeted by the bta-miR-1839 through TargetScan. Interestingly, *ESR1* was downregulated in the non-pregnant beef heifers compared to the pregnant group (Banerjee et al., 2023). In humans, *ESR1* gene expression and protein abundance were disrupted in the endometrium of patients with severe preeclampsia (Garrido-Gomez et al., 2021). Likewise, the role of *ESR1* with the cAMP signaling pathway was identified as critical in decidualization (Kaya Okur et al., 2016). Moreover, an association of genetic variants in *ESR1* has been identified with recurrent pregnancy loss in women (Pan et al., 2014; Bahia et al., 2020). Previous studies reported *ESR1* mediating the biological activity and proliferative effects of estrogen on the reproductive tissues, including ovarian follicular cells (Hewitt and Korach, 2003). Moreover, ERα (*ESR1*) knockout female mice were sterile and preovulatory follicles did not ovulate upon superovulation treatment (Hewitt and Korach, 2003). In our study, *ESR1* was associated with the activation of immune response and pattern recognition receptor signaling pathway, further supported by previous human studies (Kovats, 2015).

The SFH group heifers exhibited bta-miR-2419-5p as significantly downregulated and with low miRNA-gene connectivity (29 targets) in our study and associated with *LILRA4* identified by TargetScan. In a study on super stimulatory response in cattle, plasma miR-2419-5p was identified as downregulated in unstimulated low ovarian response heifers compared to high response heifers (Gad et al., 2020). *LILRA4* is a member of leukocyte immunoglobulin-like receptors (LILR) that regulate innate and adaptive immune functions (Hogan et al., 2012). *LILRA4* was significantly downregulated in the subfertile beef heifers at weaning compared to the heifers conceived to AI during the first breeding season (Banerjee et al., 2023). This was supported by the findings in a study on blood transcriptome in Holstein cows where *LILRA4* was downregulated in the miscarriage cow group compared to the pregnant group (Zhao et al., 2019).

The importance of miRNA function is not only for regulating adaptive and innate immune response but also for cellular proliferation, trophoblast invasion and cellular differentiation (Hayder et al., 2018), which are important for a successful pregnancy. We identified bta-let-7a-5p as downregulated in the SFH group heifers. Expression of let-7a-5p in the placenta during the first trimester has been involved with low cytotrophoblast proliferation (Smith et al., 2021). In a similar context in humans, let-7a was downregulated in the blood plasma of females with recurrent pregnancy loss compared to controls (Jairajpuri et al., 2021). In a transcriptome study with human plasma and placenta, let-7a-5p was upregulated at 11–23 weeks of gestation compared to the 6–10 weeks group (Smith et al., 2021). For further insight, we identified bta-let-7a-5p with more miRNA-gene connectivity in the SFH group (33 targets) than in the FH group (29 targets). Furthermore, *GATM* and *MXD1* genes were regulated by bta-let-7a-5p identified by TargetScan. *GATM,* a gene that encodes L-arginine and catalyzes the rate-limiting step in the synthesis of creatinine, was associated with the amino acid biosynthesis pathway in our study. This is further supported by a previous study where *GATM* is expressed during development and is imprinted in mouse placenta and yolk-sac (Sandell et al., 2003).

Apart from these miRNA-gene pairs, we identified bta-miR-574 with a high negative correlation (>0.85) with *FKBP4* and *N4BP1* in the SFH group and with *TRADD*, *HDHD2* and *HSF1* in the FH group. Bta-miR-574 exhibited a loss of gene connectivity in the SFH group. Interestingly, miR-574 was identified to be upregulated in women with preeclampsia and involved with endothelial dysfunction (Lip et al., 2018). Furthermore, miR-574 in porcine cumulus cells has been shown to suppress oocyte maturation (Pan et al., 2018). In our study, bta-miR-574 was correlated with the *FKBP4* gene that was associated with response to lipid and steroid hormone and female pregnancy pathways. In humans, *FKBP4* mRNA expression was decreased in the endometrium with endometriosis compared to controls (Yang et al., 2012). In fertile heifers, bta-miR-574 was correlated with *TRADD,* which was over-represented for apoptosis pathways. Furthermore, bta-miR-574 was correlated with *HSF1,* which was over-represented for nucleic acid transport. In previous studies, *TRADD* has been reported as regulating perinatal development and adulthood survival in mice (Dowling et al., 2019), while *HSF1* was essential for reproductive success in pre-implantation embryos (Abane and Mezger, 2010). MiRNA bta-miR-2332 was negatively correlated with *MAX* in the FH group and with *HIF1A*, *SEC63* and *COX7B* in the SFH group. *HIF1A,* in response to gonadotropins, activates steroidogenesis and cell proliferation in granulosa cells critical for ovulation (Lim M. et al., 2021), thereby exhibiting a potential role in fertility. We identified bta-miR-2478 downregulated with a loss of gene connectivity in the SFH group. Supporting our results, previous studies found that the expression of bta-miR-2478 was downregulated in bovine cumulus cells that did not cleave compared to ones that cleaved to form a blastocyst, thereby serving as an indicator of oocyte quality (Uhde et al., 2017). These miRNAs and miRNA-gene targets open up further avenues for research to be explored in relation to fertility and reproductive outcome in heifers.

From the network connectivity, we identified bta-miR-92b, bta-miR-574, bta-miR-1839, bta-miR-450b, bta-miR-2419-5p, bta-miR-874, bta-miR-2478 and bta-miR-1434-3p with a loss in network connectivity with the target genes and miRNAs, while bta-miR-2332, bta-miR-677, bta-let-7b, bta-let-7a-5p, bta-miR-1306, bta-miR-140 and bta-miR-6119-3p with a gain in network connections in the subfertile heifer group. This suggests that the loss of connectivity in heifers with low reproductive outcome is due to the rewiring of the major regulators. A potential explanation for the heifers not becoming pregnant could be attributed to the difference in the connectivity between the miRNAs and their target genes.

Some miRNAs and their targeted genes were previously reported to be associated with fertility and pregnancy outcomes in cattle and other species; herein, we reported novel miRNA-gene pairs that warrant further investigation. Combining the gene prediction of miRNAs from TargetScan and the genes differentially expressed in our previous study, we identified bta-miR-92b targeted *KLF4*, *KAT2B*, bta-miR-1839 targeted *ESR1*, bta-let-7a-5p targeted *GATM* and *MXD1*, bta-miR-1260b targeted *UBE2E1*, *SKAP2* and bta-miR-2419-5p targeted *LILRA4.* Additionally, with the PCIT network analysis approach, we identified genes with high negative correlation targeted by the miRNAs, such as *DUSP10* correlated with bta-miR-92b*, ERAS* with bta-miR-1260b, *FKBP4* with bta-miR-574 and *HIF1A* with bta-miR-2332 in the SFH group*.* Most of the previously reported studies revealed a difference in the miRNA and target gene levels during pregnancy; however, there is a possibility that these miRNAs and genes have been dysregulated for a long time without any visible symptoms until identified with pregnancy complications. Therefore, our study provides insights into the differential expression of miRNAs and genes in beef heifers as early as weaning that might play a role in predicting future reproductive performance. Thorough knowledge of the interactions will likely be needed to improve our understanding of female fertility issues and potentially develop therapeutic targets. One of the limitations of this study was the sample size, i.e., 14 heifers (7 in each group). Therefore, confirming these targets in a bigger cohort and following-up with additional time points including post-weaning, before AI and during pregnancy in heifers is required to further validate these targets and determine their potential benefit to the beef cattle industry.

## Data Availability

The datasets presented in this study can be found in online repositories. The names of the repository/repositories and accession number(s) can be found below: https://www.ncbi.nlm.nih.gov/geo/query/acc.cgi?acc&equals;GSE225854,GSE225854.
